# COVID-19 and employees’ mental health: stressors, moderators and agenda for organizational actions

**DOI:** 10.35241/emeraldopenres.13550.1

**Published:** 2020-04-20

**Authors:** Salima Hamouche

**Affiliations:** 1 Faculty of Management, Canadian University Dubai, Dubai, United Arab Emirates

**Keywords:** Coronavirus, COVID-19, Mental health, Stress, Workplace, Depression, Psychological distress, Human resource management

## Abstract

**Background:** This paper examines the impact of coronavirus COVID-19 outbreak on employees’ mental health, specifically psychological distress and depression. It aims at identifying the main stressors during and post COVID-19, examining the main moderating factors which may mitigate or aggravate the impact of COVID-19 on employees’ mental health and finally to suggest recommendations from a human resource management perspective to mitigate COVID-19’s impact on employees’ mental health.

**Methods:** This paper is a literature review. The search for articles was made in Google scholar, Web of Science and Semantic scholar. We used a combination of terms related to coronavirus OR COVID-19, workplace and mental health. Due to the paucity of studies on the COVID-19 impact on employees’ mental health, we had to draw on studies on recent epidemics.

**Results:** The identified literature reports a negative impact of COVID-19 on individual’s mental health. Stressors include perception of safety, threat and risk of contagion, infobesity versus the unknown, quarantine and confinement, stigma and social exclusion as well as financial loss and job insecurity. Furthermore, three dimensions of moderating factors have been identified: organizational, institutional and individual factors. In addition, a list of recommendations has been presented to mitigate the impact of COVID-19 on the employee’s mental health, during and after the outbreak, from a human resource management perspective.

**Conclusions: **Coronavirus is new and is in a rapid progress while writing this paper. Most of current research are biomedical focusing on individuals’ physical health. In this context, mental health issues seem overlooked. This paper helps to broaden the scope of research on workplace mental health, by examining the impact of a complex new pandemic: COVID-19 on employees’ mental health, from social sciences perceptive, mobilizing psychology and human resource management.

## Introduction

On March 11, 2020, the World Health Organization (WHO) declared coronavirus (CODIV-19) a pandemic. Which means a global disease outbreak threatening the whole planet.

CODIV-19 is an infectious disease caused by coronavirus. ‘ *Coronaviruses (CoV) are a large family of viruses that cause illness ranging from the common cold to more severe diseases such as Middle East Respiratory Syndrome (MERS-CoV) and Severe Acute Respiratory Syndrome (SARS-CoV). A novel coronavirus (nCoV) is a new strain that has not been previously identified in humans*.’ ( WHO, 2020a). They are transmitted between animals and humans. They include fever, dry cough, shortness of breath and breathing difficulties, tiredness with possible symptoms of aches and pains, nasal congestion, runny nose, sore throat or diarrhea ( WHO, 2020a)

Coronavirus is a new virus which has been discovered with its outbreak in Wuhan, China, in December 2019. Now, it has spread at a lightning speed to affect several countries. According to WHO (2020b), on March 31, 2020, this virus has reached 202 countries, areas or territories with 693,224 confirmed cases and 33,391 deaths.

Many countries have demonstrated leadership by implementing emergency measures to prevent the infection spreading. In this context, schools and university, kindergartens, cinemas, museums, restaurants have been closed, public gatherings and events have been cancelled, people quarantined, travel restrictions, close borders and cancelled flights from and to countries with a high level of contamination (e.g. China, Italy, France, Spain, US, Canada…)

Besides the negative impact on the individual, a pandemic can lead to sharp shocks to the worldwide economies and societies ( MacIntyrea, 2020; Shigemura *et al.*, 2020). According to the Organisation for Economic Co-operation and Development’s (OECD) latest Interim Economic Outlook (2020), ‘ *the coronavirus Covid-19 presents the global economy with its greatest danger since the financial crisis’. ‘Even in the best-case scenario of limited outbreaks in countries outside China, a sharp slowdown in world growth is expected in the first half of 2020 as supply chains and commodities are hit, tourism drops and confidence falters. Global economic growth is seen falling to 2.4% for the whole year, compared to an already weak 2.9% in 2019*’ ( OECD, 2020). This situation can have a negative impact on business sustainability and individual employment. In fact, this has triggered furloughs and layoffs ( World Economic Forum, 2020). Employees, in this case, need to take care of themselves, of their families and to try to maintain their job position. What about their mental health in this context?

Faced with this epidemiological catastrophe, individuals have presented anxiety-related behaviours, translated into a significant shortage of sanitizers, medical masks ( Shigemura *et al.*, 2020) and toilet paper ( Corkery & Maheshwari, 2020). Which suggests that the coronavirus is not only a physical health’s risk, but it also weighs heavily on the mental health of individuals. The best example is the tragically apparent suicide of a 37-year-old government worker, in Japan, who was responsible for looking after isolated returnees from Wuhan (China) ( The Japan Times, 2020). In China, COVID-19 outbreak has led to tremendous psychological problems that have created an emerging serious challenge for mental health services in China ( Li *et al.*, 2020)

Indeed, it seems that during a pandemic outbreak, especially in the case of an unknown new virus, individuals’ mental health issues can sometimes be largely overlooked. The objectives of the present paper were twofold. First, to examine COVID-19 impact on employees’ mental health in organizations. Secondarily, to evaluate the main organizational interventions, from human resource management perspective, which may mitigate this impact. As we write this paper, the coronavirus is spreading so fast. Considering its novelty, studies, which have investigated its impact on individuals’ mental health, are sparse. In addition, there are few studies that have examined this epidemiological catastrophe from a managerial perspective.

## Methods

Based on the classification of Grant & Booth (2009), the method used in this paper is a general literature review, which provides an examination of the recent and current literature and covers different subjects in varying levels of completeness and comprehensiveness. The subject covered in this paper is coronavirus COVID-19 that the whole world is facing while we are writing it. We examine specifically its impact on employees’ mental health, including the stressors, we explore the moderating factors as well as the possible avenues of organizational actions to mitigate the effects of COVID-19 on the employee’s mental health. The narrative form is the main characteristic of this type of review ( Grant & Booth, 2009) that we have adopted in this paper.

We searched for articles in Google scholar, Web of Science and semantic scholar using a combination of terms related to coronavirus OR COVID-19 and workplace; COVID-19 and employees’ mental health; COVID-19 and psychological distress; COVID-19 and depression, workplaces’ strategy and COVID-19. Articles were chosen according to their relevance to our research topic. We searched for articles that provided information about COVID-19’s impact on employees’ mental health, we focused on those published between December 2019 and March 2020. Biomedical articles were not selected. Our objective was to analyse articles which help to create a bridge between epidemiology, psychology and human resource management. Due to the paucity of studies on the COVID-19’s impact on employees’ mental health, we had to draw on studies on recent epidemics like SARS (Severe acute respiratory syndrome) and Ebola. We searched, in this case, for articles that link these epidemics to mental health. All the reviewed articles are included in this paper and listed in the references.

## Literature review

### COVID-19, the workplace and employees’ mental health

We examine in this paper two mental health outcomes: psychological distress and major depression that can result from a pandemic or an epidemic outbreak ( Chiu *et al.*, 2020; Lai *et al.*, 2020; Perlis, 2020; Wu *et al.*, 2005; Xiang *et al.*, 2020). Psychological distress is largely used as an indicator of mental health ( Drapeau *et al.*, 2011). It refers to a state of individual’s emotional suffering, accompanied by symptoms of depression (e.g. sadness and loss of interest) and anxiety (e.g. restlessness) ( Drapeau *et al.*, 2011; Mirowsky & Ross, 2003; Payton, 2009) and somatic symptoms like insomnia ( Drapeau *et al.*, 2011; Marchand, 2004). Psychological distress is related to a set of psychophysiological and behavioural symptoms that are distributed over a continuum of time ( Marchand, 2004). While depression is psychiatric mood disorder, characterized by persistent reduced mood and interest ( Bonde, 2008), persistent feelings of sadness, negative emotions and difficulty to cope with everyday responsibilities ( Cummins *et al.*, 2015). If not identified psychological distress may lead to major depression ( Marchand, 2004). While depression may lead to severe consequences like suicide ( Beck & Alford, 2009; Cummins *et al.*, 2015).

Psychological distress and depression are the results of an intense or a continuous stress which has not been managed, mainly due to the individual’s difficulty to cope with stressful life events ( Cummins *et al.*, 2015; Drapeau *et al.*, 2011; Marchand, 2004). The current pandemic is a source of intense stress for the whole world population.

The COVID-19 pandemic can be related to many stressors that may drain employees’ mental health, during and after this pandemic. In this section, we have made the distinction between the stressors during the coronavirus pandemic and those that can evolve after this pandemic. The distress that an individual feels is not the problem. It is rather the consequence of the problem ( Mirowsky & Ross, 2003). Therefore, it is important to understand the problem in order to be able to identify solutions which will help employees and organizations to reduce the risk of mental health issues. This is the main objective of this article.

### Stressors during the coronavirus pandemic

The main stressors during a pandemic are the 1) perception of safety, threat and risk of contagion ( Brooks *et al.*, 2020; Xiang *et al.*, 2020) ; 2) Infobesity and the Unknown ( Gao *et al.*, 2020; Garfin *et al.*, 2020) 3) quarantaine and confinement ( Brooks *et al.*, 2020; Qiu *et al.*, 2020; Wang *et al.*, 2020), 4) stigma and social exclusion ( Brooks *et al.*, 2020; Xiang *et al.*, 2020) and 5) financial loss and job insecurity ( Brooks *et al.*, 2020; Zhou *et al.*, 2020).

***Perception of safety, threat and risk of contagion.*** During pandemic, fear and panic set in. In fact, individuals’ anxiety may increase following the first death and an increased media reporting related to the number of new cases ( Rubin & Wessely, 2020). In this case, individuals are afraid about their own health and the health of the members of their family ( Bai *et al.*, 2004; Brooks *et al.*, 2020; Xiang *et al.*, 2020). The outbreak of COVID-19 itself and the control measures taken may lead to widespread fear and panic ( Zhang *et al.*, 2020a). Fear behaviours can propel the virus transmission and spread in pandemic areas ( Chan, 2014; Shultz *et al.*, 2015). For example, during Ebola, there were some cases of fearful symptomatic patients’ escape from treatment centres, concealing sick relatives at home ( Chan, 2014; Shultz *et al.*, 2015). Feeling unsafe and vulnerable to pandemics are, according to some authors, predictors of poor mental health ( Brooks *et al.*, 2018).

***Infobesity versus the unknown.*** During pandemic outbreak, individuals face an infobesity or an information overload. They become overwhelmed by the known lethality of the infection as well as the intensity of media coverage of this pandemic outbreak, which exacerbates their perception of danger ( Bai *et al.*, 2004; Garfin *et al.*, 2020; Shigemura *et al.*, 2020), increases their anxiety ( Shigemura *et al.*, 2020) and undermines their mental health. In this case, misinformation spreads faster than COVID-19.

Social media is one of the main channels providing updated information regarding COVID-19 ( Bao *et al.*, 2020; Gao *et al.*, 2020). Although it could play an important role in facilitating the communication of individuals who are quarantined with their relatives who are far away ( Brooks *et al.*, 2020), social media is not always a trusted source of information for updates about the pandemic ( Gao *et al.*, 2020). In fact, it may spread rumours or false information leading to misinformation overload ( Bontcheva *et al.*, 2013; Roth & Brönnimann, 2013), which stokes unfounded fears among many individuals. The study of Gao *et al.* (2020) showed that there was a high prevalence of mental health problems (depression and anxiety or a combination of both) which was positively associated with frequent social media exposure during the COVID-19 outbreak in Wuhan, China.

Furthermore, news coverage of a pandemic outbreak may contain an amount of conflicting information which can shake an individual’s trust ( McCauley *et al.*, 2013), creates confusion, uncertainty and increases the level of stress felt by the individual and his incapacity to cope with the intensity of the current situation. Moreover, the lack of clear information about the different levels of risks may lead individuals to imagine the worst, which exacerbates their anxiety ( Desclaux *et al.*, 2017). In fact, insufficient clear information about the pandemic and clear explanation about the necessity of quarantine have been identified as important sources of stress for individuals during the pandemic ( Brooks *et al.*, 2020).

***Quarantine and confinement.*** Quarantine refers to separating individuals (or communities) who have potentially been exposed to an infectious disease from the rest of the community ( Hawryluck *et al.*, 2004; Parmet & Sinha, 2020). It also refers to the reduction of movement of individuals who have potentially been exposed to an infectious disease ( Brooks *et al.*, 2020). COVID-19 is an infectious disease, as it spreads around the world, governments like China, Italy and many other countries have adopted draconian measures, such as imposing quarantines and travel bans, on an unexpected and unprecedented scale ( Parmet & Sinha, 2020; MacIntyrea, 2020). Although quarantines are generally established for the public good, they may result in a heavy psychological, emotional and financial burden for individuals ( Hawryluck *et al.*, 2004). In fact, individuals quarantined might experience boredom, anger and loneliness ( Xiang *et al.*, 2020). Some studies pointed out that quarantine during a pandemic, like COVID-19, is associated with poorer mental health ( Brooks *et al.*, 2020, Rubin & Wessely, 2020), with high prevalence of symptoms of psychological distress and disorder ( Wang *et al.*, 2020). This association can be worse due to the duration of the quarantine ( Brooks *et al.*, 2020). Furthermore, the study of Bai *et al.* (2004) on health care workers showed that quarantined employees were significantly more likely to report exhaustion, anxiety when dealing with febrile patients, insomnia, irritability, low levels of work performance and poor concentration. Brooks *et al.* (2020) suggested that there can be long-term negative psychological outcomes of quarantine experiences; not only for the individuals quarantined, but also for the health care system administrating the quarantine, as well as the politicians and public health officials mandating it.

***Stigma and social exclusion.*** Stigma is one of the common social consequences of a pandemic ( Xiang *et al.*, 2020). Being afraid of the risk of a potentially lethal contagious disease, people develop a form of stereotyping toward individuals associated with the epicentre of the disease, by avoiding them, blaming new disease outbreaks on them ( Desclaux *et al.*, 2017; Kinsman, 2012; Koh, 2020; McCauley *et al.*, 2013; Shigemura *et al.*, 2020; Shultz *et al.*, 2015; Xiang *et al.*, 2020) and spreading misleading rumours about them on social media ( Depoux *et al.*, 2020). Furthermore, stigma and social exclusion can be directed towards confirmed patients, survivors and their relations ( Zhang *et al.*, 2020a), and individuals who have been quarantined or who have been in contact with those who have been quarantined ( Bai *et al.*, 2004; Brooks *et al.*, 2020). Rejection, isolation, and discrimination are associated with poor psychological outcomes ( Brooks *et al.*, 2018).

Health workers are not spared from this stigma. In fact, they can even feel more stigmatization than the general public ( Brooks *et al.*, 2018). The study of Bai *et al.* (2004) showed that health care workers were more likely to feel stigmatized and rejected in their neighbourhood because of their work at the hospital. This stigmatization may lead to a high level of psychological distress and depression ( Kinsman, 2012; Zhang *et al.*, 2020a). They may suffer from it in extreme ways, for example during the Ebola outbreak there were cases neighbours throw stones at healthcare workers and chase them from their houses ( Guimard *et al.*, 1999). According to some authors, providing accurate and timely information about the disease may minimize stigmatization of health care workers ( Bai *et al.*, 2004).

***Financial loss and job insecurity.*** Pandemics lead to business disruption. The outbreak of a pandemic causes the closure of schools and workplaces ( Ferguson *et al.*, 2006), as well as the shortening of working hours ( Tyko, 2020) as measures to mitigate the severity and spread of the disease. As businesses cannot operate at their previous capacity, most of them close, which will lead to a wide spread of staff lay-offs and redundancies that will substantially decrease the level of employment ( Page *et al.*, 2006). This situation will have a negative impact on the individuals’ financial capacity due to the loss of income ( Zhou *et al.*, 2020). Financial loss can also be an issue for individuals who are quarantined, since they are not able to work or to maintain their professional activities, often without the prior ability to plan for this eventuality long-term, with potential long-lasting effects ( Brooks *et al.*, 2020). The study of Zhang *et al.* (2020b) showed that individuals who stopped working due to Covid-19 outbreak reported worse health and distress. Likewise, the study of Mihashi *et al.* (2009) showed, in the case of SARS infection, that income reduction highly predicts psychological disorder with odds of 25.0. In addition, some authors identify inadequate insurance and compensation as one of the risk factors for poor mental health ( Tam *et al.*, 2004).

Furthermore, the impact of the pandemic outbreak on businesses would significantly increase an individual’s feeling of job insecurity, which can have a negative impact on the mental health of employees who are affected by the organizational reforms of closure and reduction of working hours during COVID-19. The negative effect of job insecurity has been widely documented in literature on mental health in the workplace ( Strazdins *et al.*, 2004; Virtanen *et al.*, 2002).

### Stressors post Coronavirus

Studies suggest that some stressors that have evolved during pandemic outbreaks have long-lasting effects ( Brooks *et al.*, 2020). Which means that they remain even after the disappearance of this pandemic. At the time of writing this paper, COVID-19 is still present. Thus, it is not possible to accurately identify its effects on individuals’ mental health after its disappearance. However, if we build on recent literature related to COVID-19, literature related to previous pandemics and epidemics such as SARS, some predictions can be made concerning the potential stressors post COVID-19 which may have a negative impact on employees’ mental health. In this case, besides posttraumatic stress disorder related to the recovery from a life threatening physical illness ( Wu *et al.*, 2005), it seems that stigma, financial loss and job insecurity may have a long-lasting effect after COVID-19.

 It appears, according to the study of Siu (2008), that stigma persists in the post SARS era. The author argued that SARS victims were still experiencing stigmatization up to four years after the SARS outbreak, which maintained their social isolation, increased their level of stress and worsened their mental health. The participants of this study have reported that they have encountered stigmatization and isolation in their workplace after SARS, from their colleagues and even from their employers.

Furthermore, financial loss and job insecurity may be considered as long-lasting stressors related to COVID-19. In fact, COVID-19 has led to business disruption of some companies that will need time to recover from the financial consequences of this pandemic. This may create a spillover effect on the employment market with a potential long-lasting negative impact on employees’ finances which may lead to a negative impact on their mental health. Indeed, it appears that those disasters that result in major financial issues for individuals are associated with high levels of severe and persistent psychological effects ( Norris *et al.*, 2002).

## Moderators: what are the mitigating or aggravating factors of COVID-19’s effects on employees' mental health

Three main dimensions of moderating factors that may mitigate or aggravate COVID’s impact on employees’ mental health are examined in this paper: organizational factors, institutional factors and individual factors.

### Organizational factors

Organizational factors are related to occupational role, occupational safety and health management as well as teleworking.

***Occupational role.*** The exposure to the pandemic vary based on the working environment and the employee’s occupational role ( Bai *et al.*, 2004; Brooks *et al.*, 2018). Therefore, its impact on employees’ mental health is supposed to vary as well. In this context, besides their work overload ( Maunder, 2004), health care employees have a very high exposure to the virus since they are in constant contact with the general public, which makes their occupation high risk in terms of mental health, especially during a pandemic ( Bai *et al.*, 2004; Chen *et al.*, 2005; Huang & Zhao, 2020; Huang *et al.*, 2020; Ho *et al.*, 2020; Koh, 2020; Maunder *et al.*, 2006; Maunder, 2004; Wu *et al.*, 2009; Xiang *et al.*, 2020; Zhu *et al.*, 2020). During the COVID-19 in China, the vice minister at the National Health Commission announced on February 14, 2020, that six health workers have died from the new coronavirus and more than 1,700 have been infected ( CNA, 2020). Maunder (2004) pointed out that being a nurse, having contact with SARS and having children is associated with a high level of psychological distress.

***Occupational safety and health management*.** Employers have the responsibility to protect their employees and to ensure a workplace free from hazards that may physically harm them or cause their death. The current situation caused by COVID-19 is challenging for organizations all over the world. In this context, managers should work closely with human resource practitioners and health institutions in order to develop a safety and health plan which will prevent the risk of contagion and coronavirus spread within the organization. Organization’s policies play an important role in this context in minimizing the spread of the virus. For this purpose, they need to follow the guidelines of health officials, of their country’s government and of the World Health Organization ( Benson & Dix, 2009). They need to educate and train their employees about prevention behaviours and to provide the required protection material for those who need to be present in the workplace (e.g. Masks, Sanitizers, social distancing…). They also need to post prevention guidelines (e.g. wash hands, avoid touching eyes, nose and mouth) ( Ramesh *et al.*, 2020), and to allow telework if possible ( Benson & Dix, 2009). Having clear preventive measures in the workplace will build trust which will help to reduce employees’ level of stress. They will feel protected and supported by their employer ( Brooks *et al.*, 2018).

***Teleworking*.** In order to control the risk of CODIV-19’s spread , many employees in different countries were required to stay at home away from their workplace, triggering teleworking practices.

Teleworking is the best solution to maintain the company’s operations while ensuring the health and safety of employees during a pandemic, and to secure an income for the quarantined employees ( Greer & Payne, 2014). However, it can lead to a negative impact on employees’ mental health, mainly because it increases social isolation ( Gajendran & Harrison, 2007; Henke *et al.*, 2016; Tavares, 2017), which is associated with a high risk of psychological distress and depression. In fact, being away from his workplace and colleagues, an employee can feel isolated. Furthermore, teleworking can cause employees to work more hours because the boundaries between private and professional life are not clear ( Gajendran & Harrison, 2007; Henke *et al.*, 2016; Tavares, 2017). In addition, the level of stress may increase with the presence of children at home since schools are closed.

### Institutional factors

In this paper institutional factors refer to the governmental programs that aim to support employees financially and psychologically during and after the pandemic.

Governmental programs, mainly financial security programs, help to reduce the incidence of psychological disorder during pandemics ( Mihashi *et al.*, 2009). They are important factors to take into consideration in future strategies for mass isolation during pandemics ( Mihashi *et al.*, 2009). For example, countries that have a high level of COVID-19 infection such as France, Spain and the UK have implemented emergency packages that include direct payouts to employees; loans and guarantees for companies to mitigate the economic impact of the pandemic ( Mallet & Dombey, 2020), which will help individuals to maintain an income during the pandemic.

Furthermore, the presence of an effective mental health system can mitigate the consequences of COVID-19 on individuals’ mental health ( Qiu *et al.*, 2020; Zhang *et al.*, 2020a; Zhou *et al.*, 2020). Shultz *et al.* (2015) argue that the absence of mental health and psychosocial support systems, paired with an absence of well-trained mental health professionals, have increased the risks of psychological distress during Ebola. Prioritization of investment like the Pandemic Emergency Financing Facility launched by the World Bank Group aids the development of sustainable health systems ( Bitanihirwe, 2016). In fact, during and immediately after the pandemic outbreak, psychosocial support is crucial for quarantined people and health workers ( Zhang *et al.*, 2020a). During the COVID-19 outbreak in China mental health services have been provided using various channels like hotlines, online consultations, online courses ( Gao *et al.*, 2020; Liu *et al.*, 2020) and telemental health services ( Zhou *et al.*, 2020).

According to Xiang *et al.* (2020), mental health care for patients and health workers affected by COVID-19 has been under-addressed. The authors argued that although emergency psychological crisis interventions based on the SARS outbreak has been launched on January 26, 2020, in China, to provide psychological support during COVID-19, most health professionals working in isolation units and hospitals have not received training in how to provide mental health care. Xiang *et al.* (2020) suggest an urgent development of timely mental health care, based on the creation of multidisciplinary mental health teams established by health officials; provide a clear communication with a regular update about COVID-19 and the set-up of secure services to offer psychological counselling using electronic devices and applications (e.g. Smartphones and WeChat); and regular screening for depression, anxiety and suicidal tendencies should be performed for COVID-19 patients as well as health workers. In this context, public health officials should develop a nationwide strategic planning for psychological first aid through telemedicine ( Qiu *et al.*, 2020) and provide effectively clear messages that will help individuals to have an accurate understanding of the situation ( Brooks *et al.*, 2020) .

### Individual factors

In this paper, individual factors encompass sociodemographic factors (gender, age and education), the history of the individual’s mental illness, and the perception of physical health vulnerability.

There are no specific studies which investigate this moderating role of these factors in the relationship between COVID-19 outbreak and employees’ mental health. However, it is possible to make some predictions based on workplace mental health’s literature. In fact, research has shown that women are more prone to depression than men ( Bonde, 2008; Read & Gorman, 2011) and they have greater psychological vulnerability to stress, which suggest that they may react more intensely to stress compared to men, in the case of a pandemic ( Brug *et al.*, 2004; Zhu *et al.*, 2020). In addition, the study of Braunack-Mayer *et al.* (2013) showed that pregnant women, and those with young children, are more concerned about becoming infected or transmitting the virus to others; which may suggest that they might be more stressed than men and other women who are not in the same situation. Conversely, one study showed that being a male was a predictive factor for the onset of psychological disorders during SARS ( Mihashi *et al.*, 2009). Furthermore, it appears that older adults are more likely to be at high risk of mental health issues, mainly because of the high rate of mortality among them during COVID-19 ( Yang *et al.*, 2020), which make them vulnerable physically and psychologically. Generally, they are lonely with little social support (no children or their children have left home) ( Yang *et al.*, 2020), and they have limited access to the online mental health services due to the lack of technological skills, which might significatively undermine their mental health ( Yang *et al.*, 2020). Moreover, education is supposed to have a buffer effect because more educated people have better cognitive skills which may help them to cope with the consequence of any disability ( Brug *et al.*, 2004; Drapeau *et al.*, 2011; Mihashi *et al.*, 2009) In addition, a history of mental illness is a risk factor during pandemics ( Brooks *et al.*, 2020). An individual’s perception of their physical health, if poor, is also associated with higher stress and psychological morbidity ( Tam *et al.*, 2004) It is also the case if they have a history of chronic illnesses ( Wang *et al.*, 2020).

## Suggestions and recommendations: What can be done from a human resource management perspective to mitigate the outcomes of COVID-19 on employees’ mental health during and after the pandemic outbreak

Based on the identified stressors which may explain the potential negative effects of COVID-19 outbreak on employees’ mental health and moderating factors that may mitigate or aggravate these effects, we have developed a list of considerations and recommendations for workplaces, mainly for managers and for human resource management practitioners. It appears that mitigation measures are needed during and after a pandemic in order to reduce its potential negative effects on an individual’s mental health ( Brooks *et al.*, 2020). In this context, we suggest that organizations should develop a short- and long-term organizational plan, based on the following recommendations:

### Optimize communication and transparency

Managers in collaboration with human resource management professionals need to develop a communication plan, which clearly presents the decisions related to the business continuity plan of the organization during the pandemic ( Smith *et al.*, 2007). Furthermore, managers should maintain continuous communication with their employees whether they are physically present or not in the workplace ( Greer & Payne, 2014). Moreover, employers should involve employees in the preparation of the post pandemic business plan, which will reduce employees’ level of stress, foster positive attitude and reinforce team cohesion. In fact, decision latitude has been largely documented as a buffer of the stressors that may undermine employees’ mental health ( Karasek, 1979).

Communication is also crucial following the pandemic, in order to reduce employees’ uncertainty and their level of stress. In this context, a communication plan should be developed in order to provide clear information to employees about what will happen after COVID-19, what are the main actions that will be taken to resume organizational operations, and the potential impact of these actions on employees’ work. Indeed, providing clear and transparent information about the organization’s future plans may reduce the fear of the unknown.

### Prevention of stigma

Stigmatization can be minimized by providing accurate and timely COVID-19 information ( Bai *et al.*, 2004) and training ( Brooks *et al.*, 2020) to employees and managers during and after the pandemic outbreak. Furthermore, organizations should develop or reinforce workplace policies that address stigma prevention. For example, the development of a zero-tolerance policy (anti-discrimination) ( Stewart, 2018) is a valuable tool to protect employees, prevent stigma, and enhance health and wellbeing in the workplace.

### Training

Training is also essential during and after the pandemic. It is considered as a protective factor against mental health issues ( Brooks *et al.*, 2018). It helps to educate employees about the necessary behaviours and their importance in the prevention of viral spread. General education about COVID-19 and the reasons for quarantine can reduce stigmatization ( Brooks *et al.*, 2020) in the workplace. Training also needs to involve managers. COVID-19 is an unexpected crisis, managers need to be coached and trained on how to properly manage it, which may reduce their level of stress. They also need to be trained on how they should manage virtual teams, considering the context of teleworking, in order to be able to support their team members. Co-development programs should be implemented in this context, to develop employees and managers’ abilities to cope with the COVID-19 impact on the workplace.

### Management of teleworking and prevention of social isolation

In order to prevent the negative outcomes of teleworking on employees’ mental health during COVID-19, organizations should develop proper strategies to support employees during organizational changes. The study of Greer & Payne (2014) put forward some strategies identified by teleworkers, that may help to overcome the challenges of teleworking. These strategies encompass continuous communication with co-workers and supervisors, during teleworking, about expectations, work progress and availability. As well as providing flexibility to the employee to organize his work schedule and priorities. Moreover, good technological equipments should be provided to employees, in order to facilitate their work and interaction with their supervisor and co-workers, and reduce their level of stress. Teleworkers also need to be trained on the utilization of technology to facilitate their work and communication while they are away from their workplace, which will reduce their level of stress ( Greer & Payne, 2014).

### Social support

Social support at work is largely documented in the literature as a protective factor against workplace mental health issues ( Karasek & Theorell, 1990). The development and implementation of mental health support and services are crucial to prevent mental health outcomes of COVID-19 ( Xiang *et al.*, 2020; Xiao *et al.*, 2020). Some studies pointed out that inadequate psychological support from the employer represents a risk factor for poor mental health ( Brooks *et al.*, 2018; Tam *et al.*, 2004). The study of Wu *et al.* (2005) showed that mobilization of resources for emotional support may enhance resilience of SARS survivors. In order to mitigate the potential negative impact of quarantine, social isolation, fear of contagion and uncertainty on employees, managers should foster a supportive environment in the workplace ( Brooks *et al.*, 2018). In this context, social support programs need to be developed during and after COVID-19, by maintaining continuous communication with employees ( Greer & Payne, 2014), for example by organizing regular virtual team meetings. Employee assistance should also be provided in this situation ( Benson & Dix, 2009), it can be through employee assistance programs which should be offered for managers and non-managers. Indeed, although managers are those who enable organizations to recover from a major crisis ( Wooten & James, 2008), they are not immune from mental health problems, they also need support from their team members ( Hamouche, 2019), by maintaining continuous contact with them.

### Development of return-to-work plan

Employers should also develop a return-to-work plan for employees who have been quarantined or was in a teleworking mode, during COVID-19. This type of plan may reduce the employees’ level of stress and the risk of mental health issues. In this case, the employer should discuss expectations and the company’s future plans with the employee prior to his return to work. Work accommodations and a gradual return-to-work can be considered, in this context ( Durand *et al.*, 2014), if needed by the employee who has been quarantined or has suffered from a mental health issue during the pandemic.

## Contribution of the present paper

The present paper is a literature review which examines the impact of coronavirus COVID-19 on employees’ mental health, mainly psychological distress and depression. It presents a review of the main stressors during and post pandemic, as well as the potential moderating factors in the relationship between COVID-19 and employees mental health. Three dimensions of moderators have been reviewed: organizational, institutional and individual dimensions. The goal of this paper is to enrich the understanding of COVID-19’s impact on employees’ mental health, and to suggest avenues for organizational actions from a human resource management perspective, during and post COVID-19 in order to mitigate its effects. Very few articles have examined COVID-19 from psychological and managerial perspectives. This paper helps to broaden the scope of research on workplace mental health, and to provide some insights for managers and human resource management practitioners.

### Practical implications for organizations

COVID-19 crisis is unprecedented in terms of infectiousness, how quickly the illness spread to different countries, impacting the world’s economy. Companies are not all equipped to cope with this pandemic, in terms of information, resources and competencies. Managers and human resource practitioners need to find ingenious solutions to maintain operations while ensuring the protection of their employees. This paper provides valuable information that helps organizations to understand the main stressors during COVID-19 and those potentially to be present after COVID-19. It also provides information about the main moderating factors that may mitigate or aggravate the impact of the COVID-19 on employees’ mental health. The recommendations presented in this paper should help the managers and human resource practitioners to develop an intervention plan for the period during and after COVID-19, to maintain an efficient and rapid continuous communication with their employees including managers and to maintain partnership of managers, human resource practitioners, health and government’s officials.

## Conclusion and future research

The novelty of the COVID-19 and its potential negative impact on employees’ mental health urge this type of review. The main goal of this paper is to provide the necessary information to prevent or mitigate the negative impact of COVID-19 on employees’ mental health. We consider that the quality of the literature reviewed in this paper helps to achieve this goal.

The contribution of the literature review should be, however, considered in light of certain limitations. First, the potential for the selection of the articles to be subjective. However, the databases used (Google scholar, web of science and semantic scholar) provide the most cited articles. Furthermore, the informative character of this paper and its main objective to provide useful information for employees and organizations do not require a systematic review of the literature. Therefore, this article is contributing with a well-condensed and a well-structured paper based on information obtained from a large literature review of the impact of COVID-19 or other pandemics on employees’ mental health. This literature review can be useful for the development of a conceptual model that can be tested empirically in future research to determine the association between the identified stressors and employee’s mental health during or post COVID-19 outbreak. Second, the studies related to COVID-19 were conducted while the pandemic is still going, which does not help to identity the real stressors post COVID-19 and to confirm the presence of a causal relationship. Future research needs to be performed in this case to explore this relationship. Moreover, future research may explore other stress factors or moderating factors that are not explored in this paper, like the history of the physical health of the individual, marital status, organization size. Finally, most of the articles highlight the vulnerability of health care workers’ mental health during and after the pandemic. Future research may explore specifically the impact of COVID-19 on health workers mental health. It may cover, in this case, other mental health outcomes like burnout.

## Data availability

### Underlying data

All data underlying the results are available as part of the article and no additional source data are required
