# Supplementary material for: COVID-19 and employees’ mental health: stressors, moderators and agenda for organizational actions
Source: Emerald Open Res. 2020 Apr 20;2:15. doi: 10.35241/emeraldopenres.13550.1 (PMC7342058; doi:10.35241/emeraldopenres.13550.1)
Supplement: Peer review history

# Peer review: Reviewer response for version 1

**DOI:** 10.21956/emeraldopenres.14621.r26947

**Published:** 2020-07-06

**Authors:** Sabine Saade

**Affiliations:** 1 Department of Psychology, American University of Beirut, Beirut, Lebanon

Hamouche S. conducted a literature review pertaining to COVID-19's contribution to workers' mental health issues. More specifically, the author was interested in the association between COVID-19 outbreak, psychological distress and depression. 

 The topic explored is certainly timely as research pertaining to the association between

 COVID-19 and employees's mental health remains scant. Given the repercussions COVID-19 could have on employees including uncertainty, disruptions, etc. publishing such a study could have significant implications for employers and employees alike. Those findings could help prevent and guide mental health efforts in workplaces.

 The article is well written and clear. The design adopted in this study appropriately supports the research question. Given that the number of studies published on the topic are not numerous, the author has adequately managed to report on the few studies that have. Relatedly, findings and conclusions were well-articulated despite the scarcity of published research on the topic. No statistical analysis was used in this study hindering my ability to comment on this aspect of the study. Studies examined were appropriately cited and data from those studies was accurately extracted. Conclusions pertaining to the association between COVID-19 and employees' mental health were adequately drawn and presented. 

 Overall, the research question has been adequately addressed and the study results could significantly contribute to the meager body of literature pertaining to this emerging topic.

 Thank you again for the opportunity to review this interesting manuscript. Based on the scientific soundness of the study and its contribution to the meager body of literature pertaining to COVID-19 and mental health issues, I would recommend for the article to pass peer review as is.

Is the work clearly and accurately presented and does it cite the current literature?

Yes

If applicable, is the statistical analysis and its interpretation appropriate?

Not applicable

Are all the source data underlying the results available to ensure full reproducibility?

Yes

Is the study design appropriate and is the work technically sound?

Yes

Are the conclusions drawn adequately supported by the results?

Yes

Are sufficient details of methods and analysis provided to allow replication by others?

Yes

Reviewer Expertise:

Mental health at work and developmental disorders

I confirm that I have read this submission and believe that I have an appropriate level of expertise to confirm that it is of an acceptable scientific standard.

# Peer review: Reviewer response for version 1

**DOI:** 10.21956/emeraldopenres.14621.r26745

**Published:** 2020-05-04

**Authors:** Saju M.D

**Affiliations:** 1 Social Work, Rajagiri College of Social Sciences, Cochin, Kerala, India

This study is a crucial and timely one in the current context of Covid-19 global pandemic. This is an interesting study and the author has collected information from number of studies. However, in my opinion, the paper has some short comings with respect to the methodology and the results. Following suggestions can be considered for the improvement.

**Title and abstract: **The study methodology can be added to the title, to clarify the readers that it is a literature review.

**Background**

 Background section content can be further categorized under different domains and sequenced to justify the major review question. E.g. Domains of stressors such as, physical, social, economic, emotional, cognitive etc. To a certain extend the important research questions remain unanswered.

**Methodology**

 Full search string and strategy is not mentioned. The search string vaguely mentioned in the methodology part does not indicate the inclusion of articles on other epidemics like, SARS, EBOLA etc., but it was included in search and data extraction. So it would be good to resolve this ambiguity.

 Even though the study does not claim as a systematic review, the criteria and process of inclusion of studies could be systematic to publish it as a literature review.

 Consider adding the following elements to the methods section

- Justification for using only three search engines
- A clear string to gauge information on the said area.
- A pictorial representation of the process of selection of the studies
- Total number of studies included from all the three search engines after the screening process.

The author has included journals, website references and other documents for the review which was not mentioned in the methodology.

**Results**

 Results could be presented in a separate heading, which is missing in the text.

 An introduction to the main results can be given at the beginning of the results section, explaining the major domains of the findings.

 Results can be more organized with the help of major themes and sub themes. Stressors and its Mental health implications on planning Organizational mental health intervention strategies, need to be discussed with the help of gathered data and published evidences; efforts have been taken, but it would be better if it is little more specific. 

 Results are spread over the entire paper. Some of the main findings can be summarised into a tabular form along with the descriptions to ensure better comprehension.

**References **

- In text citations should be cited as specified in the author guidelines.
- Please cross check the references. Some of them do not seem to follow the guidelines for presenting references.

Is the work clearly and accurately presented and does it cite the current literature?

Partly

If applicable, is the statistical analysis and its interpretation appropriate?

Not applicable

Are all the source data underlying the results available to ensure full reproducibility?

No source data required

Is the study design appropriate and is the work technically sound?

Partly

Are the conclusions drawn adequately supported by the results?

Partly

Are sufficient details of methods and analysis provided to allow replication by others?

Partly

Reviewer Expertise:

Health, Mental health, Disability, Mental illness, Workplace Mental health

I confirm that I have read this submission and believe that I have an appropriate level of expertise to confirm that it is of an acceptable scientific standard.
